# A comparative transcriptomic, fluxomic and metabolomic analysis of the response of *Saccharomyces cerevisiae* to increases in NADPH oxidation

**DOI:** 10.1186/1471-2164-13-317

**Published:** 2012-07-17

**Authors:** Magalie Celton, Isabelle Sanchez, Anne Goelzer, Vincent Fromion, Carole Camarasa, Sylvie Dequin

**Affiliations:** 1 INRA, UMR1083 SPO, 2 place Viala, F-34060, Montpellier, France; 2 INRA, MIG, F-78350, Jouy-en-Josas, France

## Abstract

**Background:**

Redox homeostasis is essential to sustain metabolism and growth. We recently reported that yeast cells meet a gradual increase in imposed NADPH demand by progressively increasing flux through the pentose phosphate (PP) and acetate pathways and by exchanging NADH for NADPH in the cytosol, via a transhydrogenase-like cycle. Here, we studied the mechanisms underlying this metabolic response, through a combination of gene expression profiling and analyses of extracellular and intracellular metabolites and ^13^ C-flux analysis.

**Results:**

NADPH oxidation was increased by reducing acetoin to 2,3-butanediol in a strain overexpressing an engineered NADPH-dependent butanediol dehydrogenase cultured in the presence of acetoin. An increase in NADPH demand to 22 times the anabolic requirement for NADPH was accompanied by the intracellular accumulation of PP pathway metabolites consistent with an increase in flux through this pathway. Increases in NADPH demand were accompanied by the successive induction of several genes of the PP pathway. NADPH-consuming pathways, such as amino-acid biosynthesis, were upregulated as an indirect effect of the decrease in NADPH availability. Metabolomic analysis showed that the most extreme modification of NADPH demand resulted in an energetic problem. Our results also highlight the influence of redox status on aroma production.

**Conclusions:**

Combined ^13^ C-flux, intracellular metabolite levels and microarrays analyses revealed that NADPH homeostasis, in response to a progressive increase in NADPH demand, was achieved by the regulation, at several levels, of the PP pathway. This pathway is principally under metabolic control, but regulation of the transcription of PP pathway genes can exert a stronger effect, by redirecting larger amounts of carbon to this pathway to satisfy the demand for NADPH. No coordinated response of genes involved in NADPH metabolism was observed, suggesting that yeast has no system for sensing NADPH/NADP^+^ ratio. Instead, the induction of NADPH-consuming amino-acid pathways in conditions of NADPH limitation may indirectly trigger the transcription of a set of PP pathway genes.

## Background

Redox homeostasis is a fundamental requirement for the maintenance of metabolism. Intracellular redox potential is determined principally by the ratio of NADH/NAD^+^ and NADPH/NADP^+^ cofactors, which are involved in about 200 reactions in *Saccharomyces cerevisiae*[1]. These metabolites are interconnected by many different pathways. Any change to intracellular redox balance therefore has far-reaching effects on the metabolic network. Redox engineering strategies thus often have wide-ranging effects on metabolism [2-4]. A better understanding of the way in which yeast efficiently adjusts its metabolic fluxes in response to genetic or environmental redox changes is therefore essential for the prediction and control of these effects.

**Figure 1:**
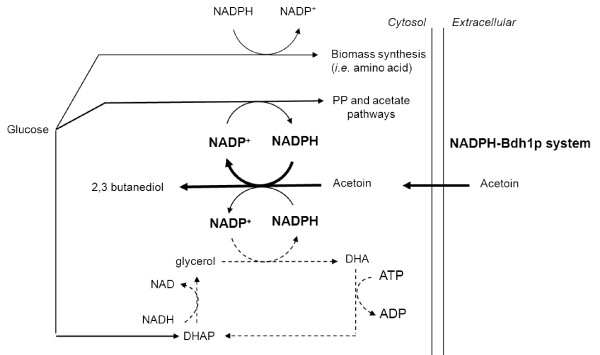
**Schematic diagram of the mechanisms involved in the response to increases in NADPH demand.** Increases in NADPH demand were imposed by adding acetoin to the growth medium of a strain overexpressing an engineered NADPH-dependent butanediol dehydrogenase (NADPH-Bdh1p). Using the *DynamoYeast* model, we previously predicted that the glycerol-DHA cycle (dashed line) acts as a transhydrogenase system, supplying additional NADPH in response to high NADPH demand [5]. DHA: dihydroxyacetone; DHAP: dihydroxyacetone phosphate.

We recently investigated the response to increases in NADPH oxidation using a biological system in which NADPH demand can be specifically and gradually modified [5]. This system involves the culture of a strain overexpressing an engineered NADPH-dependent Bdh1p enzyme in the presence of acetoin (Figure1). We analyzed the effect of four levels of NADPH demand (obtained by adding 0 to 300 mM acetoin to the medium) on intracellular flux, through the use of a constraint-based approach based on *DynamoYeast*, a dedicated stoichiometric model of *S. cerevisiae* during fermentation [5]. The pentose phosphate pathway (PP pathway) and the acetate synthesis pathway (*via* the action of the NADP^+^-dependent acetaldehyde dehydrogenase Ald6p) satisfied 80 and 20%, respectively, of the NADPH demand when this demand was increased to up to 22 times the anabolic requirement. If demand was increased still further (40 times the anabolic demand), the PP pathway was saturated and our model predicted a role for the glycerol-DHA cycle, which exchanges NADP^+^ and NADH for NAD^+^ and NADPH, at the expense of one ATP molecule (Figure1).

Despite these significant advances in our understanding of NADPH metabolism, little is known about the mechanisms regulating NADPH homeostasis. It is generally thought that the pentose phosphate pathway is controlled principally at the enzymatic level, with NADPH and ATP competitively inhibiting both the glucose-6 phosphate dehydrogenase Zwf1p and the 6-phosphogluconate dehydrogenase Gnd1p [6]. The coordinated regulation of genes involved in NADPH metabolism, including most of PP pathway genes, has been reported in conditions of oxidative stress. The activation of NADPH-dependent genes involves Stb5p, a zinc-binding factor [7], which also represses the expression of *PGI1,* encoding the phosphoglucose isomerase at the junction between glycolysis and the PP pathway. This transcription factor plays a key role in rerouting carbon flux to provide the additional NADPH required for the response to oxidative stress, as demonstrated by the greater susceptibility of the *stb5*Δ mutant to several chemicals and oxidants [7,8] and by the greater resistance to diamide of a strain overexpressing *STB5*[9]. Stb5p was also recently shown to be essential for acetaldehyde tolerance [10] and, under anaerobic conditions, to play a major role in the maintenance of basal flux through the PP pathway [11].

High-throughput experiments and metabolic modeling are powerful approaches for studying the global response of cells to environmental changes and identifying the regulatory mechanisms involved in the rerouting of the metabolic network. We analyzed the transcriptome, extracellular and intracellular metabolites and metabolic fluxes, to identify the regulatory mechanisms involved in the response of yeast cells to various increases in NADPH oxidation. We found that redox homeostasis was maintained principally through metabolic control, with regulation of the transcription of certain genes of the PP pathway occurring at higher levels of NADPH demand. We also observed a general increase in the expression of genes involved in amino-acid synthesis, particularly those involved in the synthesis of sulfur-containing amino acids, as an indirect response to the lower availability of NADPH.

## Results

### Transcriptomic response to changes in NADPH and NADH demands

**Figure 2:**
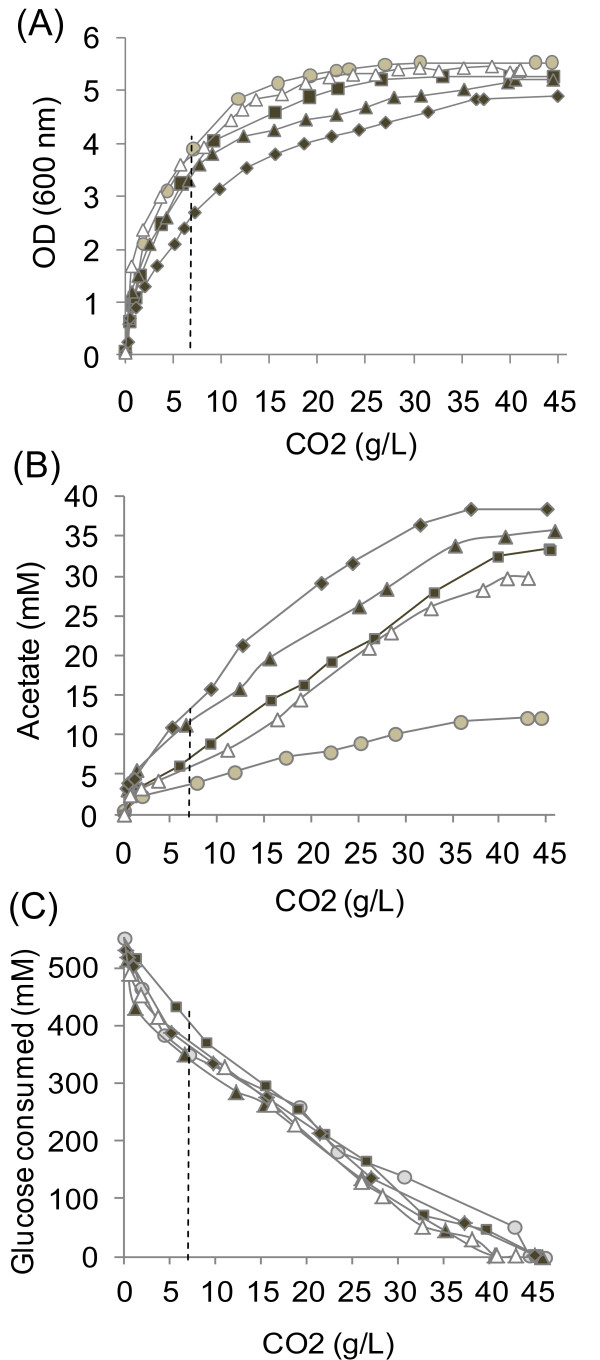
**Growth (A), acetate production (B) and glucose consumption (C) profiles during batch fermentation (2x SD, 10% glucose).** The strains were grown in the presence of 100 (square), 200 (triangle) and 300 (diamond) mM acetoin (NADPH-Bdhp), 200 mM acetoin (NADH-Bdhp, open triangle) and without acetoin (59A, circle). Growth is expressed in function of the amount of CO_2_ released determined as described in material and methods. For transcriptome and metabolome analyses, cells were sampled when CO2 reached 6 g/L (indicated by a dotted line). Complete sugar exhaustion was achieved in 36 h for 59A, in 36, 46 and 54 h for NADH-Bdhp in the presence of 100, 200 and 300 mM acetoin, and in 50 h for NADH-Bdhp in the presence of 200 mM acetoin.

We investigated the contribution of genetic control to the response to an increase in NADPH oxidation, by carrying out a microarray analysis of NADPH-Bdh cells with various levels of NADPH demand, achieved by adding 100, 200 or 300 mM acetoin to the growth medium. These conditions increase NADPH demand by factors of 8, 13 and 22, respectively, with respect to the anabolic demand [5]. We cultured the NADH-Bdh strain in the presence of 200 mM acetoin, both as a control and to assess the specificity of the effect on NADPH demand. Trancriptome analysis was performed with RNA samples extracted from cells harvested at mid-exponential growth phase when the amount of cumulative CO_2_ release was 6 g/l (Figure2A). All the strains grew at the same rate, except NADPH-Bdh in the presence of 300 mM acetoin, which had a growth rate decreased by 60% (Figure2A). At this time point, strains exposed to different degrees of change in NADPH demand differed in their acetate (a marker of NADPH metabolism) production (Figure2B) [5].

**Figure 3:**
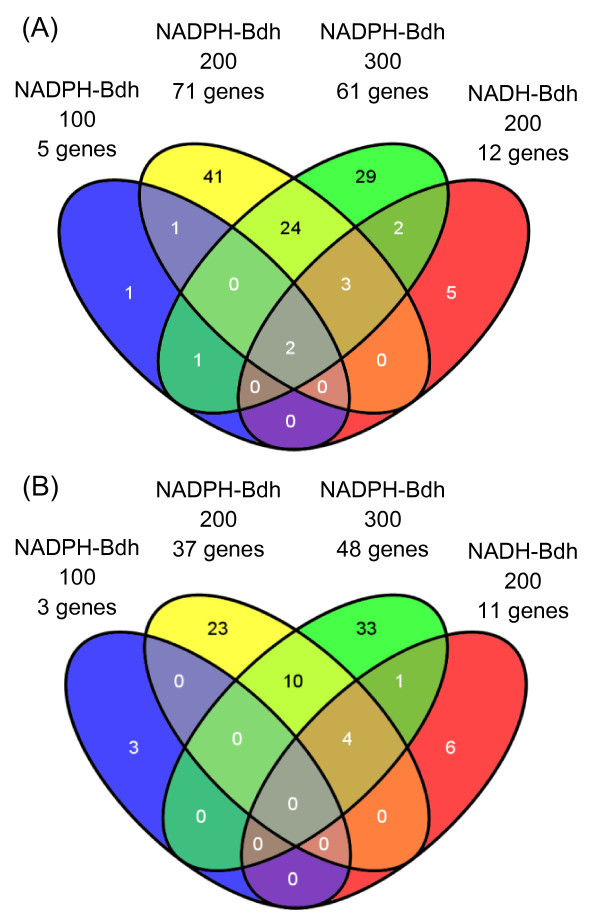
**Venn diagrams.** Upregulated (**A**) and downregulated (**B**) genes in the three NADPH demand conditions and following an increase in NADH oxidation.

We carried out multitest with a modified *t*-test, using a false-discovery rate of 5 x 10^-2^ and a threshold of a 1.5-fold change to identify genes displaying significant differences in mRNA levels between conditions in which NADPH demand was modified and control (strain 59A without acetoin) conditions (Additional file 1). The number of genes displaying differential expression clearly increased with the degree to which NADPH demand was modified (Figure3). In the presence of 100 mM acetoin, only eight genes displayed a change in expression level, whereas 71 genes were upregulated and 37 were downregulated in the presence of 200 mM acetoin and 61 genes were upregulated and 48 were downregulated in the presence of 300 mM acetoin. Changes in NADH demand had little overall effect on gene expression, as only 23 genes were differentially regulated. This suggests that the response to an increase in NADH demand to four times (at 200 mM acetoin) or in NADPH to eight times (at 100 mM acetoin) the amount required for anabolism [5] mostly involved metabolic control.

We began our study of the transcriptional response to altered NADPH demand, by carrying out a biological process gene-ontology search to characterize the NADPH response and to identify the processes involved at various levels of NADPH demand. A comparison of the gene transcript profiles of cells subjected to the modification of NADPH demand (Table 1) showed that many of the genes upregulated were involved in amino-acid biosynthesis and sulfate assimilation pathways. In particular, genes involved in the synthesis of methionine, cysteine, serine and lysine were overexpressed, whereas the expression of only two of these genes (*MET10MET17*) was modified by NADH oxidation. The upregulated genes were significantly enriched in stress-responsive genes from the seripauperin family and genes encoding ribosomal proteins. For the ribosomal protein genes at least, this may be due to differences in the phase of protein synthesis and nitrogen use, as transcriptome analysis was carried out at similar levels of CO_2_ production, but not necessarily at the same physiological stage. The decreased growth rate of NADPH-Bdh strain in the condition 300 mM acetoin did not affect the transcriptome as genes typically responding to differences in growth rate [12] were not found differentially expressed. In conditions in which NADPH demand was strongly modified, several NAD(P)H-dependent oxidoreductases were upregulated (see below).

**Table 1:** Funspec classification into biological process categories of genes up- or downregulated in response to changes in the oxidation of NADPH and NADH

	**NADH-Bdh 200 mM**	**NADPH-Bdh 100 mM**	**NADPH-Bdh 200 mM**	**NADPH-Bdh 300 mM**
f				
**Upregulated genes**				
**10**	**alcoholic metabolic bioprocess GO:0006066**			
	BDH1 ADH7	BDH1	BDH1 ADH7	BDH1 ADH7
**196**	**response to stress GO:0006950**			
		PAU24 PAU14 PAU1	PAU7 PAU24 PAU3 PAU10 PAU2 PAU11 PAU12 PAU13 PAU14 PAU15 PAU1 PAU17 PAU18 PAU23 PAU4 PAU6 MET22 PAU20 FRT1	PAU7 PAU2 PAU14 PAU17 PAU4 MET22 ZEO1 PAU20
**511**	**translation GO:0006412**			
			RPL31A RPS16B RPS17B RPL27B RPL23B RPL30 RPL24A RPL24B RPL40B RLP24 RPS31 RPS25B RPS22B RPS17A RPL15B RPL9B RPS3 RPS19A RPL25 RPS28A RPL20B RPL33A	
**41**	**methionine biosynthetic process GO:0009086**			
	MET10 MET17		MET6 MET3 MET14 MET17 MET22 MET16	MET6 MET10 MET5 MET14 MET17 MET22 MET16
**10**	**L-serine biosynthetic process GO:0006564**			
			SER3	SER3 SER33
**12**	**lysine biosynthetic process GO:0009085**			
			LYS12 LYS9	LYS12 LYS9
	**hydrogen sulfide biosynthetic process GO:0070814**			
				CYS4
**561**	**oxidation reduction GO:0055114**			
	BDH1 ADH7 MET10 ARI1 ERG3 AHP1 AAD15			BDH1 ADH7 AAD3 YDR541C SER3 MET10 ARI1 GND1 SER33 LYS12 MET5 MDH1 LYS9 AAD15 IDH2 MET16
**10**	**pentose-phosphate shunt GO:0006098**			
			SOL3 GND1	SOL3 GND1 TAL1 TKL1
**19**	**cofactor metabolic process GO:0051186**			
				MDH1 IDH2
**9**	**glucose catabolic process to ethanol GO:0019655**			
	PDC5			
**Downregulated genes**				
**8**	**fermentation GO:0006113**			
	ADH4		ADH4	ADH4
**129**	**vacuolar protein catabolic process GO:0007039**			
				PEX29 HXK1 STF2 TFS1 PGM2 DDR48 GPH1
**9**	**glucose catabolic process to ethanol GO:0019655**			
				PDC1 PDC5
**322**	**translational elongation GO:0006414**			
			ZRT1 PRY1 TPO1 AGA1 WHI5 PDR12 SAM3	THI2 ZRT1 QDR2 PRY1 IZH2 WHI5 PDR12 SAM3
**103**	**carbohydrate metabolic process GO:0005975**			
				EMI2 HXK1 PGM2 GPH1

The repressed genes included *ADH4,* which encodes an NADH-dependent alcohol dehydrogenase. This gene displayed the highest level of repression, by a factor of 5 at an acetoin concentration of 200 mM and a factor of 10 at an acetoin concentration of 300 mM. *ADH4* was also downregulated (by a factor of 3.7) in response to the modulation of NADH levels. These results suggest that the product of the *ADH4* gene is involved in redox homeostasis. Adh4p is generally thought to make use of NAD^+^, but the role, distribution within the cell and cofactor specificity of this enzyme remain unclear [13].

At an acetoin concentration of 300 mM, several genes involved in carbohydrate metabolism and alcoholic fermentation, such as *PGM2EMI2GPH1HXK1PDC1* and *PDC5,* were repressed. *PGM2* is involved in the production of UDP-glucose, a precursor of trehalose and trehalose-6-phosphate (T6P) [14], whereas *GPH1* and, probably, *EMI2* are involved in glycogen catabolism. By contrast, *PDC5* was upregulated in conditions in which NADH levels were modified.

### Pathways involved in NADPH synthesis

**Figure 4:**
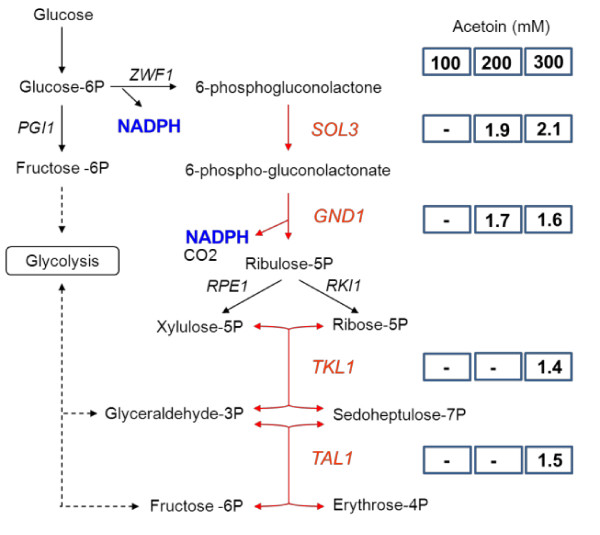
**Transcriptomic response of PP pathway genes to increases in NADPH demand.** Induction levels (fold-changes) are indicated in boxes for 100 (left), 200 (center) and 300 (right) mM acetoin. Upregulated genes are shown in red.

In our previous model-based data reconciliation analysis of the response to NADPH demand modulation, we showed that yeast cells responded to increases in NADPH demand by increasing flux through the PP and acetate pathways, which covered 80% and 20% of the NADPH demand, respectively [5]. No effect on the expression of genes involved in acetate production (*ALD* genes) or the PDH-by pass was observed, other than the repression of *PDC* genes. By contrast, several genes of the PP pathway were upregulated in response to increasing NADPH demand (Figure4). *GND1* and *SOL3* were induced when a moderate increase in NADPH demand was imposed (200 and 300 mM acetoin). In the conditions in which NADPH demand was highest (300 mM acetoin), two genes of the nonoxidative part of the PP pathway, *TKL1* and *TAL1,* were also upregulated. By contrast, the expression of *SHB17* encoding the enzyme sedoheptulose-1,7 biphosphatase recently found to be involved in riboneogenesis, a pathway that converts glycolytic intermediates into ribose-5-phosphate without production of NADPH [15], was not affected by redox perturbations.

### NADP(H)-consuming pathways

**Figure 5:**
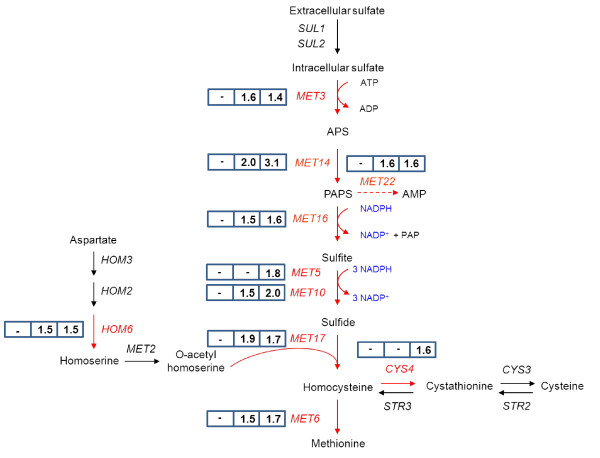
**Transcriptomic response of genes of the sulphur assimilation pathway in various NADPH demand conditions.** Induction levels (fold-changes) are indicated in boxes for 100 (left), 200 (center) and 300 (right) mM acetoin. Upregulated genes are shown in red.

One major response to increasing demand for NADPH was the activation of many genes of the sulfate assimilation pathway, which mediates the conversion of SO_4_^2−^ to methionine, *S*-adenosylmethionine (SAM) and cysteine (Figure5). This response was progressive, with the number of genes displaying changes in expression and the intensity of induction increasing with increasing NADPH demand. The entire sulfate assimilation pathway was overexpressed in the presence of 300 mM acetoin. In addition, several genes of the lysine synthesis pathway, *LYS12*, *LYS9* and *LYS2* were upregulated, as well as two genes encoding the first enzyme of the serine synthesis pathway, *SER3* and *SER33* (Table 1, Additional file 1).

Several of the downregulated genes are linked to the sulfate assimilation pathway: *SAM3,* which encodes a permease required for SAM utilization, and *TPO1*, which encodes an H^+^ transporter involved in exporting the spermidine produced from SAM. Another gene, *QDR2* encodes a multidrug transporter activated in response to the limitation of amino-acid synthesis [16].

Genes encoding several other oxidoreductases, such as *ADH7ARI1, GND1, YDR541C* (in the presence of 200 mM acetoin), *MDH1, IDH2* and *AAD15* (in the presence of 300 mM acetoin) were also upregulated in response to changes in NADPH demand. *ADH7* showed the highest level of variation of these genes. It was overexpressed by a factor of 8 in the presence of 200 mM acetoin and by a factor of up to 14 in the presence of 300 mM acetoin. *ADH7* encodes an NAD(P)H-dependent, medium-chain length alcohol dehydrogenase with broad substrate specificity thought to be involved in the accumulation of fusel alcohols, in lignin degradation and in the detoxification of furfural [17,18]. *ARI1* encoding an intermediate-subclass short-chain dehydrogenase/reductase that can use aromatic and aliphatic aldehydes as substrates and that contributes to the detoxification of furfural [19], was also strongly induced (by a factor of four) in the presence of 200 and 300 mM acetoin.

Despite the dependence of the corresponding enzymes on NADPH, *ADH7* and *ARI1* were also induced when NADH levels were modified, albeit to a lesser extent. One possible reason for this is that the changes in NADH levels also affected NADPH/NADP^+^ balance. Indeed, we previously suggested that a cycle exchanging NADPH for NADH operated in strain NADH-Bdh and that the activity of this cycle was increased by NADH oxidation, with the overall effect of decreasing NADPH availability [5]. This would account for the induction of *ADH7* and *ARI1* by changes in NADH levels.

### Rerouting metabolic flux in response to increases in NADPH demand

Fluxomics and metabolomics are powerful methods for the quantitative analysis of cell metabolism. We used ^13^ C flux analysis to estimate the distribution of intracellular carbon flux in the 59A and NADPH-Bdh strains cultured under the same conditions as for the transcriptomic analysis, but in smaller volumes (10 ml). The most striking change in flux distribution (Additional file 2) was a gradual increase in the flux through the PP pathway with increasing NADPH demand, by factors 1.6, 2.4 and 2.9 in the presence of 100, 200 and 300 mM acetoin respectively, compared to control conditions (Table 2). This confirms experimentally the progressive increase through the PP pathway flux (2.2, 3.7 and 4) previously predicted by the *DynamoYeast* constraint-based model in the same conditions [5]. We also observed a decrease in fluxes toward biomass and in fluxes toward anabolic precursors, such as cytosolic and mitochondrial pyruvate, acetyl-CoA, oxaloacetate and α-ketoglutarate, consistent with the lower growth rates in conditions of high NADPH demand (200 and 300 mM acetoin; Figure2A). Consequently, the PP pathway made a greater contribution to the formation of fructose 6-phosphate (F6P) and glyceraldehyde 3-phosphate (G3P) (Table 2).

**Table 2:** Flux distributions as a function of NADPH demand

Acetoin (mM)	0	100	200	300
Glucose uptake (mmol/gDW/h)	17.8	19.5	19.6	25.7
Glycolysis	84.7 ± 0.9	78.8 ± 1.9	69.9 ± 0.6	65.2 ± 0.6
PPP	11.4 ± 0.9	18 ± 1.9	26 ± 0.9	33.0 ± 1.4
Transketolase	5.6 ± 1.5	9.0 ± 2.5	12.2 ± 2.7	18.6 ± 4.2
Biomass (Carbohydrates)	3.3 ± 0.1	3.4 ± 0.1	2.5 ± 0.1	2.1 ± 0.1
Pyruvate decarboxylase	164.3 ± 0.1	164.8 ± 0.5	167.1 ± 0.1	167.9 ± 0.4
Pyruvate carboxylase	4.2 ± 0.8	2.7 ± 0.7	2.1 ± 0.6	2.5 ± 0.6
Import of oxaloacetate into the mitochondria	3.4 ± 0.8	2 ± 0.7	1.6 ± 0.6	2 ± 0.6
Acetaldehyde dehydrogenase	2.4 ± 0.1	3.8 ± 0.06	4.2 ± 0.1	5.6 ± 0.1
Biomass (Lipids)	0.29 ± 0.01	0.17 ± 0.02	0.15 ± 0.02	0.09 ± 0.04
Acetyl-CoA synthetase	0.30 ± 0.03	0.20 ± 0.06	0.22 ± 0.1	0.13 ± 0.09
Ethanol production	161.9 ± 0.1	161 ± 0.5	162.9 ± 0.5	162.3 ± 0.5
Glycerol production	17.8 ± 0.1	17.9 ± 0.1	14.7 ± 0.1	13.7 ± 0.1
Acetate production	2.1 ± 0.1	3.6 ± 0. 1	4.0 ± 0. 1	5.5 ± 0.1
Succinate production	0.30 ± 0.01	0.26 ± 0. 01	0.26 ± 0. 01	0.28 ± 0. 01

Other changes in flux partitioning were observed at the pyruvate and acetaldehyde nodes. At these junctions, carbons were rerouted toward the formation of acetaldehyde and acetate, whereas flux through acetyl-CoA synthase decreased. These findings suggest an increase in the contribution of NADPH acetaldehyde dehydrogenase (Ald6p) to the provision of cytosolic NADPH (Table 2) and are consistent with the increase in acetate production observed (Figure2B).

### Effects of an increase in NADPH oxidation on intracellular metabolites

**Figure 6:**
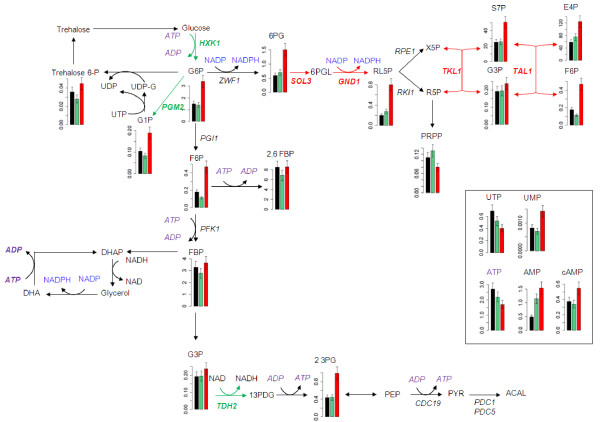
**Intracellular metabolite concentrations for the glycolysis, PP and carbohydrate pathways, following changes to NADPH demand.** Intracellular metabolite concentrations (μmol/gDW) for NADPH-Bdh in the presence of 100 (black), 200 (green) and 300 (red) mM acetoin. The genes upregulated and downregulated in the NADPH-Bdh strain in the presence of 300 mM acetoin are indicated in red and green, respectively.

We obtained a complementary view of metabolic regulation established in response to changes in NADPH demand, by determining the concentrations of 36 intracellular metabolites of the central carbon metabolism by IE-MS/MS, in the NADPH-Bdh strain cultured in the presence of 100, 200 and 300 mM acetoin, under the same conditions as for the transcriptomic analysis. We focused on the amounts of intracellular intermediates of the PP and glycolytic pathways (Figure6). The metabolite pools were clearly of similar sizes in the presence of 100 and 200 mM acetoin, but differed significantly in size in the presence of 300 mM acetoin, consistent with a major shift in metabolism to deal with these extreme conditions [5]. The intracellular concentrations of 6-phosphogluconate and of all intermediates of the nonoxidative part of the PP pathway were two to three times higher than those at lower acetoin concentrations, consistent with the increase in flux through the PP pathway. By contrast, the concentration of phosphoribosyl pyrophosphate (PRPP) decreased, consistent with the preferential rerouting of carbons through the nonoxidative part of PP pathway, at the expense of the purine and pyrimidine synthesis pathways.

We also determined the concentrations of nucleotides. The concentrations of the triphosphates decreased in response to increasing NADPH demand, by factors of 1.6 for ATP and 1.8 for UTP. By contrast, those of the monophosphate forms increased, by a factor of 2 for AMP and 1.6 for UMP. The glycolytic intermediates glucose 6-phosphate (G6P) and F6P accumulated, whereas the concentrations of G3P, 1,6-fructose-biphosphate (FBP) and fructose-2,6-bisphosphate (2,6 FBP) were similar at all levels of NADPH demand.

The UDP-glucose pyrophosphorylase (Ugp1p) is involved in the synthesis of UDP-glucose, a precursor of reserve carbohydrates, from glucose-1-phosphate (G1P). The accumulation of G1P observed in the presence of 300 mM acetoin may reflect a limitation of the flux through Ugp1p, due to a decrease in UTP availability.

An accumulation of 2-phosphoglycerate and 3-phosphoglycerate (measured as a single pool, denoted 2,3 PG) was also observed.

### Impact of changes in NADPH and NADH availability on aroma compounds

The formation of volatile compounds during fermentation is strongly influenced by redox status and may provide an overall picture of redox modifications in central carbon metabolism (CCM). We therefore investigated the impact of changes in NADPH and NADH availability on key aromatic markers (acetaldehyde, higher alcohols, acetate and ethyl esters). Acetaldehyde accumulated in large amounts, but only in response to changes in NADH levels (Table 3), whereas acetate accumulated in response to changes in the availability of either NADH or NADPH (Figure2B). The increase in acetaldehyde release therefore probably reflects limitation of the alcohol dehydrogenase reaction at low NADH availability. By contrast, increases in NADPH demand resulted in the rerouting of carbons towards acetate, to satisfy this demand (Table 2).

**Table 3:** Acetaldehyde, higher alcohols and esters produced by NADPH-Bdh and NADH-Bdh strains in response to various levels of redox disturbance

**Compound (mg/L)**	**NADPH-Bdh**				**NADH-Bdh**			
Acetoin initial (mM)	**0**	**100**	**200**	**300**	**0**	**100**	**200**	**300**
Acetoin consumed (mM)	0	100	150	245	0	100	160	164
Acetaldehyde	4.4	4.3	4.7	7.0	4.5	51.8	123.9	151.1
Ethyl hexanoate	0.13	0.09	0.08	0.03	0.15	0.12	0.06	0.05
Ethyl octanoate	0.23	0.13	0.13	0.04	0.28	0.16	0.06	0.04
Ethyl decanoate	0.16	0.02	< 0.01	< 0.01	0.13	0.06	< 0.01	< 0.01
Ethyl acetate	19.3	28.8	30.5	37.7	19.1	31.0	52.5	53.4
Isoamyl alcool	25.6	27.1	27.5	27.8	24.9	26.8	29.1	29.5
Isoamyl acetate	0.70	0.88	0.90	0.55	0.83	0.80	0.74	0.74
Isobutanol	5.5	N.D.	5.9	8.0	4.8	7.7	10.2	11.3
Isobutyl acetate	0.06	0.06	0.05	0.08	0.03	0.32	0.95	1.21
Propanol	16.3	17.1	16.5	16.3	15.3	21.1	25.1	29.3
Phenyl ethanol	16.8	26.4	N.D.	53.0	N.D.	6.8	7.6	8.5

N.D. not determined.

Increases in the oxidation of NADH or NADPH (Table 3) decreased the overall production of ethyl esters, which are generated by the condensation of ethanol and a fatty acid derivative. However, the ethyl acetate production did increase (Table 3), consistent with the observed increase in acetate concentration (Table 2, Figure2B). By contrast, acetate esters, which are produced by the condensation of a higher alcohol and acetyl-CoA, did not change significantly in abundance, with the exception of isoamyl acetate in response to the greatest level of NADPH demand. The production of fusel alcohols was increased in response to altered NADH demand, but unaffected by the modification of NADPH demand. The level of isobutanol production was 2.4 times higher in cells in which NADH levels were modified than in control cells. In line with this, the most significant effect of NADH oxidation was a large increase in isobutyl acetate production. Similarly, the synthesis of propanol doubled in response to NADH oxidation but was unaffected by increases in NADPH oxidation (Table 3). Finally, NADPH oxidation tripled phenylethanol production. The precursor of this higher alcohol is erythrose-4-phosphate, an intermediate of the PP pathway. This result is therefore consistent with an increase in flux through the PP pathway (Table 2).

## Discussion

We investigated the molecular processes involved in the maintenance of intracellular NADPH homeostasis, by studying the fluxomic, metabolic and transcriptional responses to increases in NADPH oxidation in *S. cerevisiae*. The biological system used, which was based on the overproduction of an NADPH-dependent Bdh1p enzyme and the use of acetoin as an electron acceptor, made it possible to impose unprecedented levels of redox disturbance on yeast cells, corresponding to 22 times the anabolic demand for NADPH [5].

We previously showed using constraint based metabolic flux analysis (dMBA for dynamic Mass Balance Analysis, [5]) that (i) yeast responds to increases in NADPH demand by increasing the flux through the two main NADPH-producing pathways (the PP and acetate pathways) (ii) for NADPH demands of more than 22 times the anabolic demand, the PP pathway was saturated and additional NADPH was produced through the glycerol-DHAP cycle, involving exchanges of the NADH and NADPH redox cofactors. In this study, we characterized the mechanisms involved in flux rerouting for two moderate (100 and 200 mM acetoin) and one large (300 mM) increase in NADPH, corresponding to 8, 12 and 22 times the anabolic NADPH demand, respectively. In these conditions, little effect on growth was observed only for the two highest levels of NADPH demand, consistent with a decrease in biomass synthesis due to a lack of NADPH.

### Regulatory mechanisms involved in the rerouting of carbon to NADPH-producing pathways

We combined ^13^ C-based metabolic flux, intracellular metabolite and global gene expression analyses, to investigate the way in which yeast adjusts its metabolism to respond to a high NADPH demand. We first confirmed experimentally that carbon was rerouted principally to the acetate and the PP pathways for the generation of NADPH. Flux through the PP pathway, which was estimated by ^13^ C-flux analysis, was increased by factors of 1.6, 2.4 and 2.9 in the presence of 100, 200 and 300 mM acetoin, respectively, consistent with the predictions of the *DynamoYeast* model [5].

We showed that the response of yeast to an increase in NADPH demand to eight times the anabolic demand was mediated mostly by metabolic control, as no transcriptional regulation of the genes involved in the acetate and PP pathways was observed. However, further increases in NADPH demand resulted in the induction of several PP pathway genes: *SOL3* and *GND1* in the presence of 200 mM and 300 mM acetoin and, in the presence of 300 mM acetoin, another two genes, *TAL1* and *TKL1*. These genes were probably upregulated to increase the flux capacity of the PP pathway, which was limited in these conditions. Indeed, the accumulation of 6-phosphogluconate and of all intermediates of the nonoxidative part of the PP pathway (Figure6) suggests that the activities of 6-phosphogluconolactonase or 6-phosphogluconate dehydrogenase, catalyzed by Sol3p and Gnd1p, and of enzymes of the nonoxidative part of the pathway become limiting.

Both metabolic and genetic controls are therefore required to cover a demand for NADPH more than eight times greater than the anabolic demand for this compound.

*GND1* upregulation has been observed in various situations in which the flux through the PP pathway increases [20-22]. This is not surprising, because this gene encodes a protein that catalyzes an irreversible reaction. By contrast, *ZWF1,* which encodes a protein that catalyzes the first irreversible NADPH-producing reaction of this pathway, was not transcriptionally regulated. These findings may be accounted for by differences in the cost of the proteins encoded by these genes. Indeed, a recent study in *Escherichia coli* suggested that the production of enzymes with a high cost (estimated from their abundance and molecular weight) is more tightly controlled than that of enzymes with a lower cost [23]. If the same is true in yeast, it would account for the transcriptional regulation of *GND1* rather than *ZWF1*, because the cost of Gnd1p is 10 times that of Zwf1p [24].

We initially hypothesized that the transcription factor Stb5p might be involved in the maintenance of redox homeostasis under anaerobic conditions. However, only eight of the 69 Stb5p target genes [7] (including 4 PP pathway genes) displayed differential regulation (Table 1, Additional file 1), consistent with Stb5p playing no role in the redirection of flux observed in response to increases in NADPH oxidation. This low proportion of Stb5p target genes responding to NADPH/NADP^+^ imbalance suggests that Stb5p does not sense NADPH changes directly during oxidative stress.

The repression of genes involved in the synthesis of reserve carbohydrates under conditions of the greatest NADPH demand might optimize NADPH synthesis by rerouting carbons towards the PP pathway. The same may also be true for the repression of genes involved in glycolysis and alcoholic fermentation. However, as pyruvate decarboxylase is also used to generate acetoin from acetaldehyde, the downregulation of *PDC1* and *PDC5* may result from disturbance of the acetaldehyde node. By contrast, *PDC5* was upregulated in response to altered NADH demand. Whether this may have resulted from the accumulation of acetadehyde in this condition (Table 3) remains to be explored.

### Indirect effects of changes in NADPH demand on amino-acid synthesis

The other main changes in transcription concerned the synthesis of amino acids, particularly the NADPH-consuming methionine and lysine pathways and serine synthesis, which is connected to the methionine pathway.

*MET* genes expression is normally activated by the Met4p transcription factor when the levels of these sulfur-containing compounds are low [25,26]. Sulfate assimilation requires considerable amounts of NADPH (7 moles of NADPH are oxidized to NADP^+^ to generate 1 mole of methionine from 1 mole of SO_4_^2−^). Our results therefore suggest that decreases in NADPH availability limit the flux through this pathway, resulting in low levels of sulfur-containing compound production and a global derepression of the sulfate assimilation pathway. These findings are consistent with the coordinated regulation of this pathway, which is controlled principally through feedback repression exerted primarily by cysteine [27]. The relationship between the PP phosphate pathway and the sulfur pathway has long been known [28] and is illustrated by the sensitivity to osmotic stress of a *zwf1* mutant. The connection between these two pathways does not seem to be based on transcriptional control. Indeed, no common regulator of the genes of these two pathways has been identified except Met4p, the main activator of the sulfur pathway, which can also bind *GND2*, the minor isoform of the 6-phosphogluconate dehydrogenase [29].

The upregulation of the *LYS12LYS9* and *LYS2* genes suggests that the same mechanism may operate when lysine production decreases. On the other hand, *SER3* and *SER33* encoding the first enzyme of the serine synthesis pathway, were also upregulated despite the description of this pathway as NADH-dependent [30]. However, as the biosynthetic reactions involving 3-phosphoglycerate, including those contributing to serine synthesis, constitute a highly branched network connecting the purine, thiamine, histidine and methionine biosynthesis pathways, the upregulation of these genes may be an indirect consequence of a decrease in the intracellular concentration of cysteine.

In addition, the repression of several genes linked to the sulfate assimilation pathway (*SAM3, TPO1, QDR2*) may reflect a need for the cells to conserve methionine, as the synthesis of this compound is probably compromised by the higher demand of NADPH.

### Effects of redox alteration on the synthesis of aroma compounds

We observed distinct effects of redox alteration on the production of fermentative aroma compounds, depending on the cofactor (NADH or NADPH) and on the class of aroma compounds. Indeed, increases in the oxidation of NADH and NADPH decreased the production of ethyl esters but did not alter the production of acetate esters. Changes in esters profile could reflect a decrease in the availability of acetyl-CoA [31], a precursor of these molecules, as suggested by the lower level of flux through acetyl-CoA synthase (Table 2). This suggests that the alcohol-*O*-acetyltransferase responsible for catalyzing this reaction may be less sensitive to acetyl-CoA concentration than the esterases involved in ethyl ester synthesis.

On the other hand, although the synthesis of phenyl ethanol increased in response to altered NADPH demand, consistent with increased flux through the PP pathway, the synthesis of other fusel alcohols (isobutanol, propanol) was specifically increased in response to NADH alterations. Isobutanol is synthesized from valine, which is produced from α-acetolactate, a compound also involved in acetoin synthesis. We suggest that the synthesis of acetoin by the PDC route is favored in conditions of acetaldehyde accumulation (as described by Heux *et al*. [32]), and that α-acetolactate is rerouted towards the production of valine, isobutanol and isobutyl acetate. A key mechanism underlying this remodeling of flux may be the increase in *PDC5* gene expression observed in response to NADH oxidation (Table 1). *PDC5* encodes a protein involved in the decarboxylation step of the Erhlich pathway. The greater production of butanol and propanol may therefore be explained by increased *PDC5* expression.

### The energy problem and the glycerol-DHA cycle

Analysis of the metabolic response to an NADPH demand 22 times greater than anabolic demand revealed a metabolic shift that we attributed to saturation of the PP pathway [5]. This conclusion is consistent with the intracellular metabolite profiles obtained in this study. Moreover, a previous constraint-based metabolic flux analysis suggested that a glycerol-DHA cycle exchanging NADH for NADPH was activated in these conditions [5]. The intracellular metabolite profile revealed energetic inconsistencies (Figure6), consistent with the hypothetical operation of this cycle. Indeed, the ATP and UTP pools were markedly smaller in the presence of 300 mM acetoin than in the presence of the other two concentrations of this compound. AXP concentration is one of the major factors controlling the regulation of the glycolysis and carbohydrate pathways [14,33]. The accumulation of glycolytic intermediates may result from a decrease in phosphofructokinase activity due to a decrease in ATP availability or from an increase in glycolytic flux. An increase in glycolytic flux was actually previously observed [5] in the presence of 300 mM acetoin (25.7 mmol/gDW/h instead of 19.5 mmol/gDW/h for lower −100 and 200 mM- acetoin concentrations).

Flux toward acetaldehyde, *via* pyruvate decarboxylase, increased, at the expense of the reactions supplying the TCA pathway. The NADPH imbalance results in the rerouting of the carbon atoms of acetaldehyde for the production of acetate *via* Ald6p. The marked accumulation of acetate when the cells were incubated in the presence of 300 mM acetoin (Figure2B) probably resulted from a lower level of flux through acetyl-CoA synthase, resulting from both ATP limitation and a smaller acetyl-CoA requirement for lipid biosynthesis. Consistent with the limited production of acetyl-CoA, the production of acetate esters and fatty acids from this precursor decreased in response to increases in NADPH demand.

## Conclusion

This study highlights the remarkable robustness of yeast to changes in NADPH demand, based on efficient mechanisms for the reprogramming of metabolic flux to produce large amounts of NADPH. In line with previous studies [22,34], we observed that fluxes are controlled by both metabolic and transcriptional regulation. However, the contribution of these mechanisms varies in function of the NADPH demand. Metabolic control was involved in the adaptation to increases in NADPH demand to up to eight times the anabolic demand. At higher levels of NADPH demand, transcriptional regulation of several genes also contributes to the rerouting of carbons towards the PP pathway.

## Methods

### Strains and culture conditions

The strains used in this study were derived from the *S. cerevisiae* haploid strain 59A [35]. Strains NADH-Bdh and NADPH-Bdh were obtained by overexpressing the *BDH1* gene encoding the NADH-dependent 2,3-butanediol dehydrogenase (Bdh1p) or a mutated version of this gene [36] encoding an NADPH-dependent Bdh1p [5]. Overexpression was achieved by replacing the chromosomal *BDH1* promoter with the *TDH3* promoter and, for NADPH-Bdh, by replacing the native ORF with the mutated ORF.

*S. cerevisiae* strains were maintained and grown at 28 °C on YPD medium (1% Bacto yeast extract, 2% Bacto peptone, 2% glucose). An aliquot of each culture was mixed with glycerol to a final concentration of 20%, frozen and stored at −80 °C.

### Fermentation medium and growth conditions

Batch fermentations were performed in one-liter bioreactors equipped with fermentation locks for the maintenance of anaerobiosis, at 28 °C, with continuous stirring (500 rpm). Fermentation experiments were carried out in 2X SD minimal medium (13.4% yeast nitrogen base without amino acids, 100% glucose). The SD minimal medium was supplemented with 1.25 mg/l ergosterol, 0.164 g/l Tween 80, and 0.35 mg/l oleic acid, to satisfy the lipid requirements of the yeast cells during anaerobic growth. Acetoin was added at various concentrations (from 0 to 300 mM) at the start of fermentation, as previously described by Celton *et al.*[5]. Bioreactors were inoculated to a final OD_600_ of 0.05, with cells from a YPD preculture in 50 ml of medium in a 250 ml flask, which had been incubated at 28 °C, with shaking. Fermentations were performed at 28 °C. CO2 release was determined by automatic measurements of fermentor weight loss at 20-minute intervals [37]. Fermentation experiments were performed in triplicate.

### Analytical methods

Yeast growth was monitored by measuring the optical density of the culture at 600 nm (OD_600_). Glucose and acetate were determined by high-performance liquid chromatography (HPLC) with an Aminex HPX-87 H ion exchange column. Volatile compounds were determined by GC-FID analysis, with a headspace autosampler and a BP20 (SGE).

### Microarray analysis

Gene expression was analyzed from three independent cultures of each studied strain: 59A, 59A NADH-Bdh 200 mM, 59A NADPH-Bdh 100 mM, 200 mM and 300 mM. Aliquots of 1x10^9^ cells were sampled from the fermentation medium when CO2 production reached 6 g/l/h. Cells were collected by centrifugation at 1000 g for 5 min at 4 °C and the cell pellets were washed with DEPC-treated water and frozen in methanol at −80 °C. Total RNA was extracted with Trizol reagent (Gibco BRL, Life Technologies) [38] and purified with the "RNA Cleanup kit" (Qiagen). We checked the quantity and quality of the extracted RNA by spectrometry (NanoDrop 1000, Thermo Scientific). The DNA microarrays were manufactured at the Biochip platform of Toulouse–Genopole, on dendrislides [39], with 70-mer oligonucleotides covering 99% of the yeast genome (Operon Inc.; a list of the corresponding genes is available from http://biopuce.insatoulouse.fr/oligosets/). Fluorescent cDNAs were synthesized from 5 μg of total RNA with the CleanUP System (Promega). Labeled cDNA was purified with the Pronto Purification kit (Promega). Microarrays were hybridized in static conditions for 16 h in 42 °C in a hybridization chamber (Corning), with the Pronto Universal Hybridization Quick kit.

### Transcriptomic data acquisition and statistical analysis

Hybridization signals were detected with a GenePix 4000B laser scanner (Axon Instruments). Array images were acquired and quantified with integrated GenePix software version 3.0. Statistical analysis was done with R.2.11.1 software [40]. The limma package [41-43] was used to normalize the microarray data (by the print-tip-loess method for normalization within the array followed by the quantile method for normalization between arrays). Differential expression between two sets of experimental conditions was detected by carrying out a modified *t*-test, with a significant threshold of *p* < 5 x 10^-2^, applying the Benjamini and Hochberg false discovery rate to correct for multiple testing [44]. Only genes displaying a fold-change of at least 1.5 (positive or negative) in expression level were considered. Genes displaying differential expression were grouped according to Gene Ontology (GO) process terms, with the database for annotation, visualization and integrated discovery (DAVID), beta version 6.7 [45]. The complete data set is available through the Gene Expression Omnibus database (accession number GSE34810).

### Extraction and determination of intracellular metabolites

Sampling from two independent replicates of each studied strain (NADPH-Bdh 100 mM, 200 mM and 300 mM acetoin) and carried out when CO_2_ production reached 6 g/l/h. Cells (0.5 to 1.95 x 10^8^) were added to 5 ml of methanol solution (40% vol/vol) to quench their metabolism. They were then collected by centrifugation (15000 g, 1 min, -10 °C) and stored at −80 °C. Extraction and quantitative analysis by isotope dilution mass spectrometry using uniformly ^13^ C-labeled cell extracts as internal standards [46] were carried out at the MetaToul Platform, Toulouse. Ethanol (5 μl of a 75% v/v solution) was added to the cell pellets and metabolites were extracted by incubating the mixture for three minutes at 80 °C. The samples were cooled on ice and centrifuged at 15000 g for 10 minutes. Ethanol was removed by evaporation and the metabolites were resuspended in 250 μl ultra-pure H_2_O and centrifuged at 20000 g for two minutes. Intracellular metabolites were analyzed by high-pressure anion exchange chromatography in a Dionex ICS 2000 machine (Dionex, USA) equipped with an AS11 analytical column (2 x 50 mm). Mass spectra were acquired with a QTrap 4000 electrochemical detector (Applied Biosystems) equipped with a Sciex Turbo V MSD (Toronto) for ESI electrospray ionization and MRM (Multiple Reaction Monitoring) in detector mode.

### ^13^ C-flux analysis

Cells were grown in penicillin vials filled with 10 ml 2X SD minimal medium supplemented with 1.25 mg/l ergosterol, 0.164 g/l Tween 80, 0.35 mg/l oleic acid and with 100 mg.l^-1^ of a mixture of labeled (40% 1-^13^ C-glucose) and unlabeled glucose and 1.4 mg.l^-1^ of NH4Cl as the sole nitrogen source. At an OD_600_ of 3.0, corresponding to mid-exponential growth phase, the concentration of extracellular metabolites (glucose, glycerol, organic acids) was measured in the supernatant (these data will be used to constrain the stoichiometric model, as described below). Cells were harvested and hydrolyzed overnight with 6 M HCl for the determination of amino acids labeling patterns. One glucose derivative (glucose pentacetate) and two amino-acid derivatives (ethyl chloroformate (ECF) and dimethyl formamide dimethyl acetal (DMFDMA)) were analyzed by GC-MS [47], as previously described by Gombert *et al*. [48]. From the raw GC-MS data, the summed fractional labeling (SFL) of each fragment was calculated as follow: SFL = 100 × [(1.*m*1 + 2.*m*2 + …. + n.*m*n) × (*m*0 + *m*1 + *m*2 + … + *m*n)^-1^, with *m*o the fractional abundance of the lowest corrected mass and *m*_i_ > 0 the abundance of molecules with higher corrected masses. The labelling data are available in request.

The data set used for ^13^ C-flux analysis included the 25 SFLs calculated from labeling experiments and 22 measured fluxes, including the drain of metabolic intermediates to biomass and the formation of seven metabolites, giving a total of 47 items of experimental data. The distribution of carbon in central carbon metabolism (CCM) was estimated with the metabolic model described by Gombert *et al.*[48], modified to take into account features specific to fermentation [49]. The network used is described in additional file 2. Flux calculations (60 reactions) were carried out with Matlab 7, as previously described [47,48]. Differences between experimental and simulated SFLs and between experimental and simulated fluxes were minimized by an iterative procedure. Calculations were carried out 100 times and the fluxes reported are the means of the convergent solutions (Additional file 2).

## Competing interests

The authors declare that they have no competing interests.

## Authors’ contributions

MC carried out the fermentation experiments, the transcriptomic and the fluxomic experiments, analyzed the data and drafted the manuscript. IS participated in the design of the study and performed the statistical analysis. AG and VF participated in the analysis of the data and helped to revise the manuscript. CC helped to analyze the flux and metabolic data, participated in the conception of the study and helped to draft the manuscript. SD conceived the study, participated in its coordination and helped to draft the manuscript. All authors have read and approved the final manuscript.

## Supplementary Material

Additional file 1 **Differentially expressed genes involved in the response to increases in NADPH and NADH oxidation.** The differentially expressed genes involved in the response to increases in the oxidation of NADPH (100, 200 and 300 mM acetoin) and NADH (200 mM acetoin) are shown. Click here for file

Additional file 2 ^**13**^** C-flux analysis:*S. cerevisiae* metabolic network, flux distribution in response to acetoin additions (0, 100, 200, 300 mM) and measured SFL and experimental data.** (A) Reactions of the metabolic network used for ^13^ C-flux analysis. (B) Fluxes estimated by ^13^ C-flux analysis. (C) Measured SFL. (D) Specific growth rate, glucose uptake rates and yield coefficients used in ^13^ C flux. Click here for file
