# Supplementary material for: A comparative transcriptomic, fluxomic and metabolomic analysis of the response of Saccharomyces cerevisiae to increases in NADPH oxidation
Source: BMC Genomics. 2012 Jul 17;13:317. doi: 10.1186/1471-2164-13-317 (PMC3431268; doi:10.1186/1471-2164-13-317)
Supplement: Additional file 2 — 13 C-flux analysis:S. cerevisiae metabolic network, flux distribution in response to acetoin additions (0, 100, 200, 300 mM) and measured SFL and experimental data. (A) Reactions of the metabolic network used for 13 C-flux analysis. (B) Fluxes estimated by 13 C-flux analysis. (C) Measured SFL. (D) Specific growth rate, glucose uptake rates and yield coefficients used in 13 C flux. [file 1471-2164-13-317-S2.doc]

Additional file 2

1. ^13^C-flux analysis: *S. cerevisiae* metabolic network and flux distribution in response to acetoin additions (0. 100. 200. 300 mM).
2. Reactions of the metabolic network used for ^13^C-flux analysis

**Glucose uptake**

U1: GLC <-> G6P

% EMP-pathway

E1: G6P <-> F6P

E2: F6P <-> G3P + G3P

E3: G3P <-> PEP

E4: PEP <-> PYRCYT

**PP-pathway**

PP1: G6P <-> CO + P5P

PP2: P5P + P5P <-> S7P + G3P

PP3: S7P + G3P <-> F6P + E4P

PP4: P5P + E4P <-> F6P + G3P

PP5: F6P + P5P <-> S7P + E4P

PP6: P5P + G3P <-> P5P + G3P

**Ethanol. acetate and glycerol formation**

P1: PYRCYT <-> ACA + CO

P2: ACA <-> ETH

P3: ACA <-> ACE

P4: G3P <-> GLYC

P5: AKG <-> OHG

**Formation of AcCoA in the cytosol**

ACOA1: ACE <-> ACCOACYT

**Anaplerotic reaction (cytosolic)**

AN1: PYRCYT + CO <-> OAACYT

**TCA-cycle**

ACOA2: PYRMIT <-> ACCOAMIT + CO

TC1: OAAMIT + ACCOAMIT <-> ICIT

TC2: ICIT <-> AKG + CO

TC3: AKG <-> SUC + CO

TC4: FUM <-> SUC

TC5: FUM + FUM <-> OAAMIT + OAAMIT

TC6: OAAMIT <-> FUM

**Transports**

TR1: OAAMIT <-> OAACYT

TR2: ACCOACYT <-> ACCOAMIT

TR3: PYRCYT <-> PYRMIT

**Threonine / Serine / Glycine metabolism (all enzymes assumed to be cytosolic)**

AA1: G3P <-> SER

AA2: SER <-> GLY + C1

AA3: OAACYT <-> THR

AA4: THR <-> GLY + ACA

**Malic enzyme (oxaloacetate decarboxylation. mitochondrial)**

ME1: OAAMIT <-> PYRMIT + CO

**Drain of intermediates to macromolecules**

B1: G6P <-> G6POUT

B2: P5P <-> P5POUT

B3: E4P <-> E4POUT

B4: G3P <-> G3POUT

B5: PEP <-> PEPOUT

B6: PYRMIT <-> PYRMITOUT

B7: PYRCYT <-> PYRCYTOUT

B8: OAACYT <-> OAACYTOUT

B9: AKG <-> AKGOUT

B10: ACCOACYT <-> ACCOACYTOUT

B11: ACCOAMIT <-> ACCOAMITOUT

B12: SER <-> SEROUT

B13: GLY <-> GLYOUT

B14: C1 <-> C1OUT

B15: THR <-> THROUT

**Excreted Products**

PE1: ETH <-> ETHOUT

PE2: ACE <-> ACEOUT

PE3: GLYC <-> GLYCOUT

PE4: SUC <-> SUCOUT

PE5: OHG <-> OHGOUT

PE6: AKG <-> AKGOUT

PE7: PYRCYT <-> PYRCYTOUT

**CO2-evolution**

PE8: CO <-> COOUT

1. Fluxes estimated by ^13^C-flux analysis

| **Acetoin** | **0 mM** | | **100 mM** | | **200 mM** | | **300 mM** | |
| --- | --- | --- | --- | --- | --- | --- | --- | --- |
|  | Mean | S.D. | Mean | S.D. |  | Mean | S.D. | Mean |
| **AA1** | **0.29** | 0.01 | **0.24** | 0.01 | **AA1** | **0.29** | 0.01 | **0.24** |
| **AA2** | **0.14** | 0.01 | **0.12** | 0.01 | **AA2** | **0.14** | 0.01 | **0.12** |
| **AA3** | **0.38** | 0.01 | **0.32** | 0.01 | **AA3** | **0.38** | 0.01 | **0.32** |
| **AA4** | **0.09** | 0.01 | **0.07** | 0.01 | **AA4** | **0.09** | 0.01 | **0.07** |
| **ACOA1** | **0.30** | 0.03 | **0.20** | 0.06 | **ACOA1** | **0.30** | 0.03 | **0.20** |
| **ACOA2** | **1.37** | 0.17 | **1.00** | 0.08 | **ACOA2** | **1.37** | 0.17 | **1.00** |
| **AN1** | **4.21** | 0.79 | **2.68** | 0.74 | **AN1** | **4.21** | 0.79 | **2.68** |
| **B1** | **3.88** | 0.02 | **3.17** | 0.04 | **B1** | **3.88** | 0.02 | **3.17** |
| **B10** | **0.29** | 0.01 | **0.17** | 0.02 | **B10** | **0.29** | 0.01 | **0.17** |
| **B11** | **0.45** | 0.02 | **0.36** | 0.01 | **B11** | **0.45** | 0.02 | **0.36** |
| **B12** | **0.15** | 0.01 | **0.12** | 0.01 | **B12** | **0.15** | 0.01 | **0.12** |
| **B13** | **0.23** | 0.01 | **0.19** | 0.01 | **B13** | **0.23** | 0.01 | **0.19** |
| **B14** | **0.14** | 0.01 | **0.12** | 0.01 | **B14** | **0.14** | 0.01 | **0.12** |
| **B15** | **0.30** | 0.01 | **0.25** | 0.01 | **B15** | **0.30** | 0.01 | **0.25** |
| **B2** | **0.21** | 0.01 | **0.17** | 0.01 | **B2** | **0.21** | 0.01 | **0.17** |
| **B3** | **0.21** | 0.01 | **0.17** | 0.01 | **B3** | **0.21** | 0.01 | **0.17** |
| **B4** | **0.12** | 0.01 | **0.09** | 0.01 | **B4** | **0.12** | 0.01 | **0.09** |
| **B5** | **0.39** | 0.01 | **0.32** | 0.01 | **B5** | **0.39** | 0.01 | **0.32** |
| **B6** | **1.35** | 0.09 | **1.05** | 0.05 | **B6** | **1.35** | 0.09 | **1.05** |
| **B7** | **0.00** | 0.01 | **0.00** | 0.01 | **B7** | **0.00** | 0.01 | **0.00** |
| **B8** | **0.40** | 0.01 | **0.32** | 0.02 | **B8** | **0.40** | 0.01 | **0.32** |
| **B9** | **0.79** | 0.08 | **0.58** | 0.03 | **B9** | **0.79** | 0.08 | **0.58** |
| **E1** | **84.7** | 0.9 | **78.7** | 1.9 | **E1** | **84.7** | 0.9 | **78.7** |
| **E2** | **92.1** | 0.3 | **90.6** | 0.6 | **E2** | **92.1** | 0.3 | **90.6** |
| **E3** | **169.6** | 0.3 | **168.8** | 0.6 | **E3** | **169.6** | 0.3 | **168.8** |
| **E4** | **169.2** | 0.3 | **168.5** | 0.6 | **E4** | **169.2** | 0.3 | **168.5** |
| **ME1** | **2.31** | 0.77 | **1.17** | 0.74 | **ME1** | **2.31** | 0.77 | **1.17** |
| **P1** | **164.3** | 0.1 | **164.8** | 0.5 | **P1** | **164.3** | 0.1 | **164.8** |
| **P2** | **162.0** | 0.1 | **161.0** | 0.5 | **P2** | **162.0** | 0.1 | **161.0** |
| **P3** | **2.42** | 0.03 | **3.79** | 0.06 | **P3** | **2.42** | 0.03 | **3.79** |
| **P4** | **17.8** | 0.00 | **17.93** | 0.01 | **P4** | **17.8** | 0.00 | **17.93** |
| **P5** | **0.00** | 0.01 | **0.00** | 0.01 | **P5** | **0.00** | 0.01 | **0.00** |
| **PE1** | **162.0** | 0.1 | **161.0** | 0.54 | **PE1** | **162.0** | 0.1 | **161.0** |
| **PE2** | **2.13** | 0.00 | **3.59** | 0.00 | **PE2** | **2.13** | 0.00 | **3.59** |
| **PE3** | **17.8** | 0.0 | **17.9** | 0.0 | **PE3** | **17.8** | 0.0 | **17.9** |
| **PE4** | **0.30** | 0.01 | **0.26** | 0.01 | **PE4** | **0.30** | 0.01 | **0.26** |
| **PE5** | **0.00** | 0.01 | **0.00** | 0.01 | **PE5** | **0.00** | 0.01 | **0.00** |
| **PE6** | **0.02** | 0.01 | **0.02** | 0.00 | **PE6** | **0.02** | 0.01 | **0.02** |
| **PE7** | **0.31** | 0.01 | **0.24** | 0.01 | **PE7** | **0.31** | 0.01 | **0.24** |
| **PE8** | **176.2** | 0.7 | **183.0** | 1.4 | **PE8** | **176.2** | 0.7 | **183.0** |
| **PP1** | **11.38** | 0.94 | **18.0** | 1.9 | **PP1** | **11.38** | 0.94 | **18.0** |
| **PP2** | **1.72** | 1.47 | **2.87** | 2.17 | **PP2** | **1.72** | 1.47 | **2.87** |
| **PP3** | **3.79** | 0.31 | **6.01** | 0.62 | **PP3** | **3.79** | 0.31 | **6.01** |
| **PP4** | **5.65** | 1.50 | **8.98** | 2.51 | **PP4** | **5.65** | 1.50 | **8.98** |
| **PP5** | **2.07** | 1.45 | **3.14** | 2.26 | **PP5** | **2.07** | 1.45 | **3.14** |
| **PP6** | **22.4** | 23.1 | **9.39** | 29.3 | **PP6** | **22.4** | 23.1 | **9.39** |
| **TC1** | **0.92** | 0.16 | **0.67** | 0.08 | **TC1** | **0.92** | 0.16 | **0.67** |
| **TC2** | **0.92** | 0.16 | **0.67** | 0.08 | **TC2** | **0.92** | 0.16 | **0.67** |
| **TC3** | **0.12** | 0.11 | **0.06** | 0.08 | **TC3** | **0.12** | 0.11 | **0.06** |
| **TC4** | **0.18** | 0.11 | **0.20** | 0.08 | **TC4** | **0.18** | 0.11 | **0.20** |
| **TC5** | **8.15** | 30.34 | **0.02** | 0.04 | **TC5** | **8.15** | 30.34 | **0.02** |
| **TC6** | **16.5** | 60.7 | **0.24** | 0.09 | **TC6** | **16.5** | 60.7 | **0.24** |
| **TR1** | **-3.42** | 0.78 | **-2.04** | 0.74 | **TR1** | **-3.42** | 0.78 | **-2.04** |
| **TR2** | **0.01** | 0.03 | **0.03** | 0.05 | **TR2** | **0.01** | 0.03 | **0.03** |
| **TR3** | **0.41** | 0.77 | **0.88** | 0.77 | **TR3** | **0.41** | 0.77 | **0.88** |
|  |  |  |  |  |  |  |  |  |

Fluxes are expressed in mmol/100 mmol glucose. ^13^C flux analyses were carried out 100 times for each experimental condition and the fluxes reported are the means of the convergent solutions from the 100 calculations.

1. Measured SFL

1. Specific growth rate. glucose uptake rates and yield coefficients used in ^13^C flux

| Acetoin (mM) | 0 | 100 | 200 | 300 |
| --- | --- | --- | --- | --- |
| Biomass (g) | 2.9 | 2.55 | 2.65 | 2.17 |
| Growth rate (h-1) | 0.4 | 0.36 | 0.23 | 0.15 |
| Glucose uptake rate (mmol/gDW/h) | 17.8 | 19.5 | 19.6 | 25.7 |
| Y_s(ethanol)_ (mol/mol) | 1.7 | 1.7 | 1.7 | 1.6 |
| Y_s(glycerol)_ (mmol/mol) | 182 | 184 | 161 | 95 |
| Y_s(acetate)_ (mmol/mol) | 21 | 36 | 44 | 48 |
| Y_s(pyruvate)_ (mmol/mol) | 3 | 2.4 | 3.7 | 4.2 |
| Y_s(succinate)_ (mmol/mol) | 3.1 | 2.6 | 3 | 3.5 |
